# How Can We Address What We Do Not Measure? A Systematic Scoping Review of the Measurement and Operationalization of Social Determinants of Health Research on Long-Acting Reversible Contraceptive among Adolescents in the US

**DOI:** 10.3390/adolescents3020018

**Published:** 2023-03-30

**Authors:** Catherine Poehling, Margaret Mary Downey, Anwei Polly Gwan, Sarah Cannady, Olivia Ismail

**Affiliations:** 1 School of Social Work, University of Southern Mississippi, Hattiesburg, MS 39401, USA; 2 School of Social Work, Tulane University, New Orleans, LA 70118, USA; 3 Department of Obstetrics, Gynecology and Women’s Health, University of Minnesota, Minneapolis, MN 55455, USA; 4 Upstream USA, Boston, MA 02109, USA

**Keywords:** gender equity, health equity, health disparities, adolescent health, social determinants of health, long-acting reversible contraceptives, LARC, intrauterine device, IUD, implant

## Abstract

Teen pregnancy is often considered an adverse health outcome that accentuates gender inequities, diminishes opportunities, and jeopardizes the safety of adolescent and young adult birthing people. Long-Acting Reversible Contraceptives (LARC) have been hailed as a panacea for teen pregnancy. However, adolescents and emerging adults intersect with multiple assaults on their health and well-being due to gender inequity and racism. To establish equitable care, it is imperative to discern all barriers that influence their reproductive autonomy. This study evaluates the measurement, operationalization, and quality of research conducted on adolescents and emerging adults that analyzed the use of LARC within the social determinant of health framework (SDOH) in the US. SDOH were assessed using the Dahlgren and Whitehead model, and reports were analyzed using a modified version of the Joanna Briggs Institute (JBI) Critical Appraisal tools. Nineteen articles were included in this study. Researchers found the insufficient measurement of race, ethnicity, sexuality, and gender among studies on LARC and SDOH in adolescents and emerging adults. Future studies must measure a full range of identities in data collection to generate knowledge on the impact of SDOH and LARC use among diverse populations.

## 1. Introduction

The impact of social, racial, and economic inequities on the health outcomes of adolescents and emerging adults capable of becoming pregnant is a critical concern for health providers and advocates. Previous literature has documented the impact anti-black racism, and prejudice practices have on access to quality care and, thus, maternal health and birth outcomes [1–3]. Black-birthing people are three times more likely to die from pregnancy-related causes than White-birthing people [4–8]. In addition, they are more likely to face pregnancy-related morbidities, including hypertension, diabetes, and hemorrhage [9,10]. Despite this, Black birthing people are almost twice as likely to face an unintended pregnancy compared to their White counterparts, further putting them at increased risk for poor pregnancy outcomes [11].

The high prevalence of unintended pregnancy rates and maternal mortality in Black-birthing people can be attributed to poor healthcare quality, barriers to timely healthcare, structural anti-Black racism, and implicit biases [12–14]. Social Determinants of Health (SDOH) continue to bar birthing People of Color from equitable access to many essential resources that allow them to plan and undergo their pregnancies safely and with dignity. Since the early 2000s, Long-Acting Reversible Contraceptives (LARCs) use has become more prevalent among adolescents [15,16]. National and state-level initiatives have focused on expanding these resources to low-income individuals [17].

### 1.1. Sexual and Reproductive Health of Adolescents in the United States

According to the National Survey of Family Growth (NSFG), more than half of adolescents aged 15 to 19 years (41% female, 39% male) engaged in penile-vaginal intercourse between 2006–2019 [18]. While sexual activity rates have remained stagnant over time for adolescents categorized as female, the US teen birth rate (i.e., births per 1000 females aged 15–19 years) has steadily declined since 1991 [18,19]. In 2019, adolescent birth rates were 16.7 per 1000 [19]. Although reasons remain unclear, current evidence suggests that declines are secondary to adolescents’ increased use of contraceptives [20,21]. Nonetheless, approximately 75% of adolescent pregnancies are unintentional and account for one in six unintended pregnancies in the US overall [22].

US adolescent birth rates are significantly higher than in other western industrialized nations, and substantial racial, ethnic, and sociopolitical disparities persist [19]. Compared to Non-Hispanic, White adolescents, the birth rates for Non-Hispanic Blacks and Hispanic adolescents were two times higher. In addition, the birth rates of American Indians/Alaskan Natives (AIAN) were the highest among all ethnicities [19]. Disparities in adolescent birth rates have been linked to health inequities such as geographical location, low education level, low income (<100% FPL), and housing stability [19,23–25]. Additionally, access to comprehensive health services, especially abortion care, and restrictive health policies exacerbate disparities [26,27].

Adolescent pregnancies and subsequent childbearing have been linked to significant implications and are noted as significant societal concerns. Adolescent pregnancy is linked with increased high-school dropout rates and significantly poor maternal health outcomes (i.e., prolonged premature rupture of membranes, preeclampsia, postpartum depression, and maternal deaths) [28]. Children of adolescent parents are more likely to have lower school achievement, drop out of high school, experience more health problems, face incarceration during adolescence, give birth as an adolescent, and face unemployment as young adults [19]. Moreover, teenage pregnancy and childbearing cost approximately $9.4 billion annually, indicating a significant financial burden on the country. [19].

### 1.2. Long-Acting Reversible Contraceptives

Many public health and medical providers have recently focused on highly effective contraceptive measures to combat high adolescent pregnancy rates. LARC is a form of contraception that provides long-term pregnancy prevention without requiring actions from users. Currently, three LARCs are available in the US, levonorgestrel intrauterine devices (LNG-IUD), copper intrauterine devices, and hormonal implants.

Hormonal IUDs (e.g., name brands Mirena, Lileta, Skyla, and Kyleena) release the hormone progesterone over the course of years. As of 2023, Mirena (52 mg LNG) and Lileta (52 mg LNG) have been approved for usage for up to eight years. In comparison, Kyleena (19.5 LNG) and Skyla (13.5 mg LNG) are approved for five and three years, respectively [29]. Unlike the LNG IUDs, the Copper IUD (e.g., Paragard) does not release hormonal substances. Instead, it prevents pregnancy by causing irritation and inflammation to the uterine lining and acts as a toxin for spermatozoa. It has been approved for usage for up to ten years [30]. Lastly, the etonogestrel single rod hormonal implant (e.g., Nexplanon 68 mg) is a device placed under the skin of the inner non-dominant arm and has been approved for up to three years of usage. In the US, all three are approved for usage in nulliparous and multiparous people with uteruses [31].

LARCs are highly effective at preventing pregnancy. Compared to other alternatives, such as condoms, combined oral contraceptives (COCs), progestin-only pills (POPs), estrogen-based patches and rings, or fertility awareness-based methods, LARCs are >99% effective. The failure rate for LNG-IUDs, copper IUDs, and hormonal implants is 0.1–0.4%, 0.8%, and 0.1%, respectively [32].

### 1.3. Barriers to LARC Access for Adolescents and Young Adults

According to the Centers for Disease Control and Prevention (CDC), 10.3% of people with childbearing capacities use LARCs. Young adults (13.1%) are almost two times more likely to use LARC compared to adolescents (8.2%) [33]. More notably, adolescent (<age 20) LARC usage has only increased somewhat from <3% (from 2006–2010) to 8.2% despite efforts to promote uptake or continuation [34].

Several studies have identified barriers to accessing LARCs for adolescents. For example, a systematic review conducted by Hendrik et al. (2020) identified that despite recommendations from several medical organizations, including the American College of Obstetrics & Gynecology (ACOG), the American Academy of Pediatrics (AAP), the Society of Family Planning (SFP), and the CDC, many providers are hesitant to provide LARCs to adolescents, especially when they are nulliparous [34]. These reservations stem from misconceptions regarding LARC complications [34]. These misconceptions include an increased risk of pelvic inflammatory disease (PID) resulting in infertility, ectopic pregnancies, and pelvic pain; and an increased risk of IUD expulsion [34]. In addition, people seeking contraception face barriers to uptake, such as their lack of familiarity with LARC methods, the high costs of LARC, lack of quality healthcare access, and low parental acceptance [22,35].

Several research studies have evaluated the effects of removing such barriers to LARC uptake or continuation for adolescents. One study is the Contraceptive CHOICE Project, a large prospective study of 10,000 people capable of becoming pregnant in Missouri, ages 14–45 years [36]. People were provided LARC education and no-cost IUDs and implants [use next]. Seventy-two percent of the 1404 adolescents enrolled chose a LARC method over other contraceptive methods [36]. Teens enrolled in this study experienced lower rates of pregnancy (34.0 vs. 158.5 per 1000), birth (19.4 vs. 94.0 per 1000), and abortion (9.7 vs. 41.5 per 1000) compared to the national average in 2008 [36]. This landmark study and similar evidence demonstrate the impact of reducing adolescent barriers to LARC use and their effect on health outcomes [36,37].

### 1.4. LARCs and Disparities

The link between adolescent pregnancy and the racial, ethnic, and economic disparities impacted by childbearing is a focus for health advocates. Given the numerous benefits of LARCs, many researchers, clinicians, and politicians have advocated for state-funded LARC programs to reduce access burdens [38]. However, reproductive justice and health scholars have drawn attention to the predicaments of promoting LARC use for adolescents to advance health equity [38,39]

1. The notion that LARC can solely mitigate unintended pregnancies and thus poverty;
2. The clinical emphasis of LARC over all other forms of contraceptives;
3. The disregard of the historical association between LARC promotion and racism and eugenics [38].

#### 1.4.1. LARCs as a Means to Ameliorate Social Ailments

Many advocates herald LARC as a singular solution for pregnancy prevention due to their efficacy and perceived ease of use [40]. This overly simplistic reduction suggests that inaccessibility to effective contraceptives is the sole driver behind social and economic disadvantages. This implicates unintended pregnancies as a cause rather than a consequence of inequity, failing to fully consider the racial, gendered, structural, and economic factors contributing to unintended pregnancies [38,40,41]. As Gubrium et al. (2016) indicate, eradicating adolescent pregnancy would not eliminate barriers to higher education attainment or economic inequities [40]. This viewpoint places the blame for social inequities and the burdens of social change on the reproductive practices of birthing people, particularly adolescents [38]. Moreover, this mindset may distract from the structural inequalities that serve as the root causes of poor reproductive and maternal health outcomes [38]. This approach further perpetuates social and health inequity by focusing on individual-level behavioral interventions rather than the broader systemic and structural inequities [40].

#### 1.4.2. Reproductive Coercion and LARC Promotion

LARCs are considered first-line contraceptive options, particularly for adolescents, due to their efficacy [42]. LARC proponents maintain that healthcare providers should use directive and persuasive tactics when people choose not to use LARCs, given that in some instances, it is not in the person’s best interest for their overall well-being, as LARCs are statistically the most effective medication option for pregnancy prevention [42]. However, pregnancy and pregnancy prevention are complex, and management differs from other medical conditions or illnesses.

Additionally, the assumption that efficacy is the only factor to consider in contraceptive decision-making ignores the myriad of additional factors (i.e., partner involvement, synthetic hormonal levels, a person’s self-determination, non-contraceptive benefits, and the impact of sexual health) that affect individual decisions about their elected contraception methods [38]. Furthermore, it is impossible to discuss the efficacy, accessibility, and use of LARCs without acknowledging the deeply harmful history of reproductive coercion in family planning and specific to LARC methods. LARC methods are highly effective contraceptives touted as a panacea for lowering adolescent pregnancy rates. However, when the primary focus of any method is centered on reducing unintended pregnancy as a singular or most important factor, this can lead to LARC being prescribed or preferred in contraceptive counseling in a biased way, particularly as it applies to young people.

Providers and advocates must balance the well-intended enthusiasm for a method that may be highly effective with little to no daily involvement by the person using it while also safeguarding from unintentional or intentional bias and coercion in the ways patients are counseled on or offered contraceptive methods. While LARCs are an excellent option for some people, they do not meet the many needs of all people. The LARC first lens fails to offer support for birthing people, particularly adolescents and emerging adults, for self-determination around their reproductive capacity. It ultimately lacks support for birthing people and parents.

### 1.5. Social Determinants of Health and LARCs

**Figure 1.**
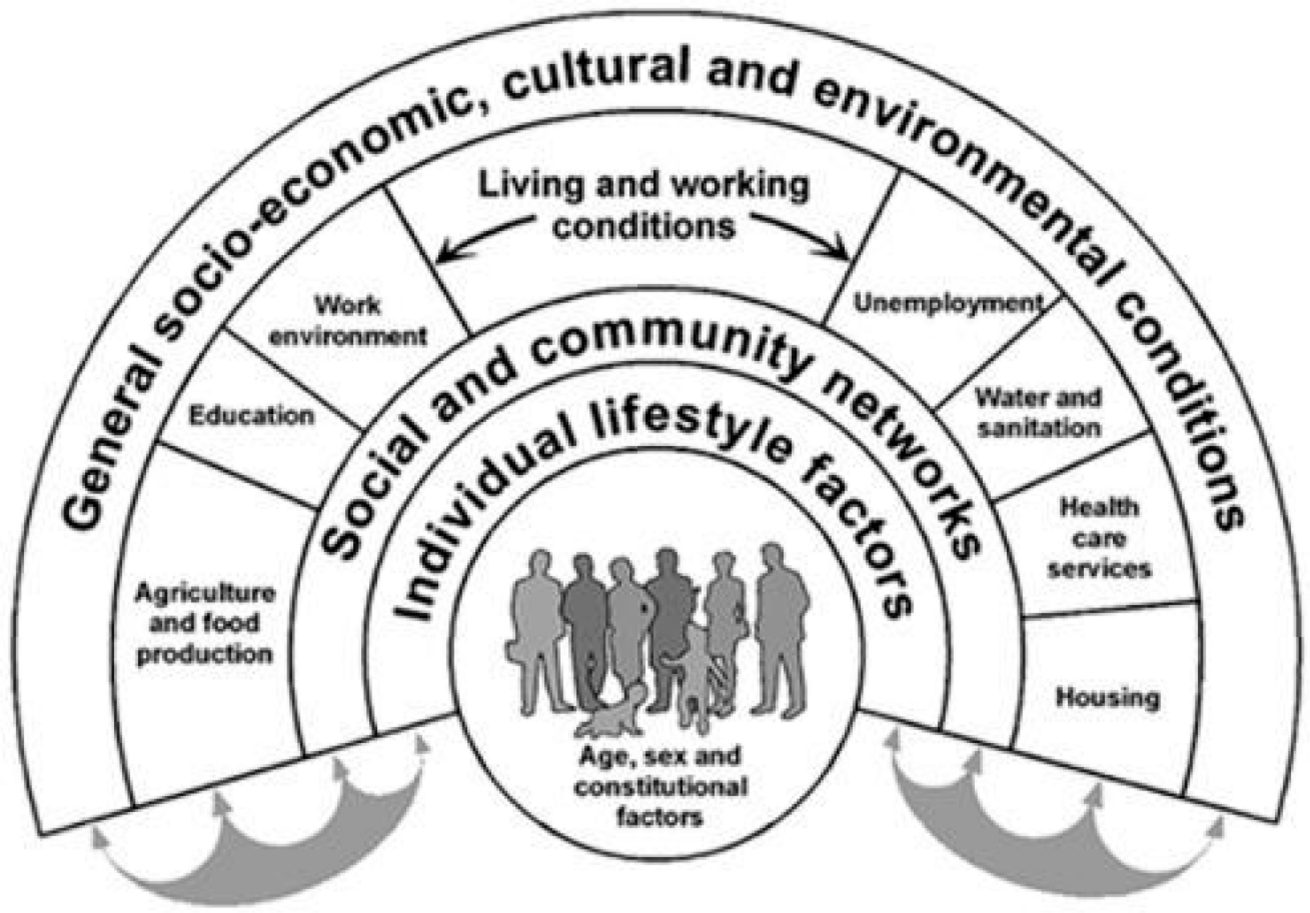
The Dahlgren and Whitehead Rainbow Model, 2021 [43] (Reprinted with permission from Ref. [43]. 2021, Dahlgren and Whitehead).

There is increasing attention to adequately understanding and addressing the social determinants of health (SDOH) that shape LARC use. The SDOH framework emphasizes factors including and above individual biology, behavior, and genetics that shape health and healthcare [43]. Such factors include living, working, learning, and playing conditions and the structural forces shaping those conditions [43]. This systematic review explores how the SDOH are measured and operationalized in the current literature on LARC use in adolescents. Specifically, the authors apply the widely cited Dahlgren and Whitehead rainbow model of the SDOH in Figure 1 to peer-reviewed research articles on studies where LARC use is a primary outcome [43].

### 1.6. Study Purpose

The primary purpose of this study is to evaluate the measurement, operationalization, and quality of research conducted on adolescents and emerging adults analyzing LARC usage within an SDOH framework in the US. Operationalization is defined as the process of precisely defining abstract concepts within research so that they can be empirically evaluated. Operationalization is especially important to research validity when measuring SDOH and healthy equity due to the abstract nature of the SDOH [44,45].

## 2. Materials and Methods

**Figure 2.**
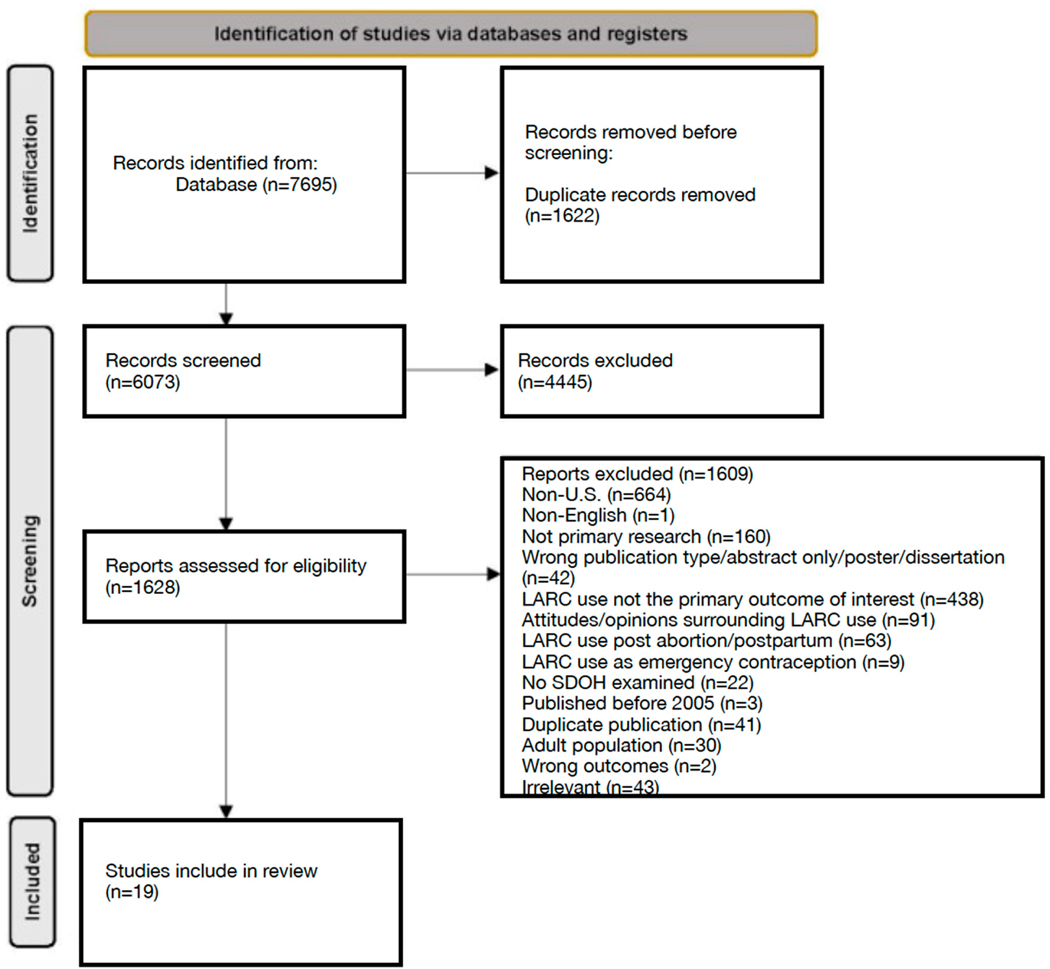
PRISMA Flow Diagram.

A systematic scoping review of the literature using the Preferred Reporting Items for Systematic Reviews and Meta-Analysis (PRISMA) format was conducted, and presented in Figure 2. A research librarian was consulted to develop a search strategy and database selection. Five electronic databases were used to search for published articles: PubMed, Embase, Web of Science, CINAHL (Cumulative Index to Nursing and Allied Health Literature), and PsycINFO beginning in December 2020. Terms to capture contraceptive methods of interest (i.e., long-acting reversible contraceptives, intrauterine device, implant) and their abbreviations, associated brand names, and synonyms (e.g., birth control) were included (Supplementary File S1). In addition, terms including social determinants of health, health equity, health disparity, and associated terms were included to develop a comprehensive understanding of the literature. Terms related to specific aspects of the SDOH framework, such as housing, healthcare, insurance, stigma, income, community, and occupation, were also included. Search terms related to contraceptive injections (e.g., Depo-Provera) were initially included but ultimately excluded from analysis as this method did not meet strict LARC criteria for reversibility after consensus from researchers.

Data collection began in December 2020 and concluded in January 2022 with directed searches of relevant studies’ references to capture additional sources. Studies published after 2005 were included as this year represented an increase in peer-reviewed, English-language scholarship published on the SDOH [46].

Researchers used this data set and further extracted studies of qualitative, quantitative, or mixed-method research designs conducted in the United States, including adolescents and young adults aged 13–28 years, as adolescents and emerging adults are closely related in development [47]. Studies were included that measured at least one SDOH. LARC use (i.e., the continuation of a LARC; initiation of a LARC, or use for the first time; or uptake, the returning to use after non-use) is a primary outcome measure. Covidence, a web-based software platform, was used to screen and manage imported references. Three reviewers (MMD, CP, AG) screened titles and abstracts, then full-text reviews. All reviewers met and independently screened five random records and discussed their decision-making processes to establish consistency; the remaining records were then divided among reviewers.

Although the study was not registered with PROSPERO, an extraction form was established a priori. Additionally, two reviewers (MMD, CP) independently assessed the quality of studies (e.g., for bias) using an adapted version of the Joanna Briggs Institute (JBI) critical appraisal checklist for analytical cross-sectional quality assessment (Appendix A). Authors used the JBI checklist to holistically understand study quality rather than to numerically score them and rule in or rule out studies. No studies were excluded to concerns about their quality [48]. Next, the authors used the extraction form, which was created a priori on individual studies to determine how they measured and operationalized the SDOH, their impact on LARC use, and identify overall themes and patterns in studies’ quality, methodological approaches, and design. Furthermore, the authors evaluated the measurement and operationalization of race, ethnicity, sex, gender, and sexual orientation via the extraction form for each study. In the US, race and ethnicity are social constructs useful for measuring systemic racism present in US society [49]. Sex or sex assigned at birth is typically operationalized via biological markers, whereas gender represents social aspects of gender expression, including identities and behaviors [50].

An outline is provided of the nineteen studies included and their characteristics (Table 1). Three reviewers (CP, APG, and SC) appraised one article and came together to discuss challenges and discrepancies and reach a consensus. Reviewers (CP, MMD, APG, and OI) independently applied the assessment tool in Google Forms in duplicate. Reviewers met during each research phase to discuss challenges and discrepancies and reach a consensus. Finally, two reviewers (CP and MMD) met to review and analyze results, grouping them into themes.

## 3. Results

### 3.1. Study Characteristics

Nineteen articles (representing 19 studies) published between 2011 and 2020 assessing social determinants of LARC usage among adolescents and young adults met inclusion criteria and were included for analysis. Sixteen studies (84%) used quantitative methodology, including retrospective (*n* = 4), prospective cross-sectional (*n* = 4), case-control studies (*n*= 1), quasi-experimental (*n* = 2), randomized controlled (*n* = 1), pre-and post-analysis (*n* = 1), ecological (*n* = 1), survey analysis (*n* = 1). Three (16%) studies used qualitative methodology. Sample sizes for studies ranged from *n* = 18 to *n* = 616,148 participants. Participants were recruited from various settings, including university-affiliated clinics or research sites, Title X clinics, and Planned Parenthood Clinics. One study extrapolated data for secondary analysis from the New York Youth Risk Behavior Surveillance System [17].

Most studies were conducted across multiple regions in the country or nationally (*n* = 4, 21%) [17,51–53] or in the Mideast region (i.e., Delaware, D.C., Maryland, New Jersey, and Pennsylvania) (*n* = 4, 21%) [54–57]. Three studies were conducted in the Southeast regions (i.e., Alabama, Arkansas, Florida, Georgia, Kentucky, Louisiana, Mississippi, North Carolina, South Carolina, Tennessee, Virginia, and West Virginia) [58–60]. Fewer studies were conducted in Rocky Mountain (e.g., Colorado, Idaho, Montana, Utah, and Wyoming; *n* = 1) [39] and Plains region (e.g., Iowa, Kansas, Minnesota, Missouri, Nebraska, North Dakota, and South Dakota; *n* = 2) [61,62].

The majority of studies (*n* = 13, 68%) explicitly evaluated all three LARC methods (Hormonal IUD, Copper IUD Non-hormonal, and subdermal contraceptive implant). Five studies (26%) evaluated only hormonal (/LNG-IUD) and non-hormonal (/Copper-IUD) LARCs only. One study defined the contraceptive methods they examined as LARCs without naming specific devices [17]. The majority of studies focused on LARC uptake (*n* = 11, 58%) by adolescent populations. Three studies (16%) explicitly assessed LARC continuation by adolescents. Two studies (11%) reviewed only LARC prevalence through a national survey database which did not explicitly collect initiation or continuation. These results are listed and described in Table 1.

### 3.2. Age

Nearly all studies (*n* = 17, 89%) specified participants’ ages, ranging from ages 13–25 (our inclusion criteria was age 13–28). One study included participants from “menarche” but did not specify the exact age minimum for inclusion [58]. One study included high school students but did not specify their ages [17].

### 3.3. Race & Ethnicity

More than half of the studies analyzed race and/or ethnicity (*n* = 13, 68%), and five measured ethnicity and race as separate constructs [17,39,61,63,64]. In one study [54], race and ethnicity were excluded to protect confidentiality, given that the study was a small focus group. Meanwhile, eight studies reported operationalizations of combined race/ethnicity or only race or only ethnicity categories [51,53–56,60,62,65]. Twelve studies reported participants who identified as White (i.e., Caucasian, non-Hispanic White, White) [17,39,51,53–55,60–65], eleven reported including participants who identified as Black or African-American (i.e., Black, African-American, non-Hispanic Black) [17,39,51,53–55,60–63,65], and thirteen reported including participants who identified as Hispanic and/or Latino (i.e., Hispanic or Latino, Hispanic, Latino, Latina) [17,39,51,53–56,60–65]. Only four studies explicitly reported on their inclusion of Asian and/or Pacific Islander participants, one on American Indian/Alaskan Native and Native Hawaiian [39,63–65].

Notably, one study [64] specified the breakdown of Asian-identified participants (i.e., predominantly Filipino and Japanese) and Pacific Islanders (i.e., Micronesian, Marshallese, and Samoan), a reporting practice that was not specified elsewhere in the literature. As the authors noted, the study’s location in Hawai’i and the sociopolitical categories around identity influenced data collection and reporting. Nine studies reported an additional “Other” category for race [39,51,53–55,61–64]. Operationalization of race and/or ethnicity in four studies was derived a priori from standardized tools, including the National Survey of Family Growth (*n* = 1), Youth Risk Behavior Surveillance (*n* = 1), and Electronic Medical Health systems (*n* = 2).

### 3.4. Gender, Sex, and Sexuality

All studies reported, in some terms, the sex or gender of participants. Of all the studies reviewed, 100% (*n* = 19) used the terms female and/or male to refer to participants. Some studies used the terms women or women and men in addition to reporting on females and/or males. No studies provided sources for the determination of gender operationalization. While no studies directly reported on the sexuality of participants, one article [64] excluded participants with same-sex partners from the analysis of LARC counseling and LARC use.

### 3.5. Social Determinants of Health

Table 2 summarizes the results of the SDOH factors authors found in this review.Nineteen studies (100%) examined factors in the age, sex, and constitutional tier of the Dahlgren and Whitehead rainbow model [17,39,51–67]. Five studies (26%) of the studies included measured individual lifestyle factors (e.g., substance use, sexual activity, and behaviors) [51,53,60,64,67]. Seven studies (37%) examined factors in the social and community networks tier (e.g., marital status, partner’s opinions and support of contraception use, religious affiliation, having social support, and the influence of social networks) [51,53,56,57,60,61,64]. Of the nineteen studies reviewed, all (100%) examined participants’ living and working conditions (e.g., having access to health insurance, salary, federal poverty level, or level of education) [17,39,51–67]. Seven studies (37%) explicitly examined general socio-economic, cultural, and environmental conditions (e.g., state Medicaid expansion, public LARC education initiative) [17,39,51,52,56,61,62].

## 4. Discussion

This study draws from the first and second authors’ larger study on measuring and operationalizing the social determinants of LARC use in the US in adults 18 years or older [68]. Consistent with previous findings that focused on SDOH among adults in the US, this study found systematic issues with the measurement and operationalization of race, ethnicity, sexuality, and gender [68]. Studies included in the current study did represent greater regional geographic representation by including Hawai’i.

### 4.1. Race and Ethnicity

Researchers must acknowledge the importance of racism and ethnocentrism, not only race and ethnicity, as factors that shape health status [69] and use person-centered ways to measure and report on participant identity and experiences of identity. Moreover, research on LARCs and SDOH among young people must also acknowledge the intersections of classism, gender, ageism, racism, and ethnocentrism. For example, many young Black, Indigenous, and other people of color who can become pregnant are scrutinized based on overlapping, reinforcing stereotypes of low-income, young people of color as irresponsible, sexually permissive, and prone to risky behavior, including sexually risky behavior. Mann (2013) notes how community health centers have focused on preventing pregnancy in Latina youth in the name of addressing the problematic sexual behaviors of an at-risk population, at the expense of inclusive, comprehensive interventions that might empower youth healthcare patients and their providers to confront structural inequities shaping their reproductive lives [70].

Limited operationalization and reporting on race and/or ethnicity can inhibit accurate reporting of health inequities due to confounding between race and ethnicity and other variables such as class and nationality [71]. Other guidelines that may improve the quality of SDOH and LARC use research in adolescents and young people include:

- The purposeful study design (e.g., noting the limitations of using racial categories;
- A hypothesis-driven analysis (e.g., not assuming race is a driving factor relevant to the study hypothesis);
- Not pathologizing or medicalizing race (e.g., not using white as a reference group, which can normalize the idea that non-white groups are “other”) and;
- Acknowledging intersectional identities (e.g., examining models within racial groups [72].

These techniques may be essential for research with adolescents and young adults, as the language around identity is shifting and dynamic and may be different for adolescents and young adults than other age groups (e.g., use of the terms Latinx and Latine) [71].

One study in this review [64] acknowledged their operationalization of race and ethnicity as a limitation, noting that “the demographic data of race and ethnicity were collected from electronic health records, which may not represent the self-identified racial identity of all patients. For example, while 23% of persons in Hawai’i identify as being of mixed race, the electronic health record permits only one race/ethnicity identification per patient” (p. 7) [64]. In line with recent calls for medical research to acknowledge the problematic and harmful legacy of racial hierarchies (often justified in and through self-proclaimed medical science), we see the previous language as an example of how scholars can engage with demographics critically. Additionally, a minority of studies in our review used qualitative or mixed methods. Increasing the number of rigorous qualitative or mixed-methods studies on the social determinants of LARC use among young people may improve our understanding of youth patients’ lives as these methods (e.g., interviewing, focus groups) more readily allow participants to discuss their identities in their own terms, with the developmental stages of adolescence and emerging adulthood in mind [73].

### 4.2. Gender, Sex, and Sexuality

One issue we noted throughout this review was the conflation of sex and gender as constructs, threatening construct validity and precision of findings [74]. ACOG recognizes health disparities related to systemic discrimination against gender minorities [75]. However, none of the studies identified data collection that would allow for expansive identity outside of the binary of male and female. Meanwhile, the Institute of Medicine promotes expansive measurement for gathering demographic data on gender and sexual identity [76]. Failing to measure or report on sexual orientation and gender-diverse people who use LARCs prohibits researchers, practitioners, and policymakers from accounting for nuanced ways that SDOH influences the accessibility of LARC uptake and use.

### 4.3. Social Determinants of Health

The authors suggest that based on the results of this review, there is growing literature on SDOH and LARC in adolescents and young adults. Given the sensitivity of contraceptive use to social factors and the importance of addressing SDOH to achieve health equity, this literature base is an encouraging one for public health. Meanwhile, research on LARC uptake and use in adolescents and young adults systematically fail to measure many socioeconomic, environmental, and cultural barriers to accessing or choosing LARC. Without capturing critical information such as family support, insurance status, policy factors, transportation, healthcare providers, and staff-level factors, it is impossible to clearly understand the impacts of SDOH on adolescent and young adult decision-making and access to LARC for pregnancy prevention and reproductive autonomy.

### 4.4. Future Research

Future research can refine and expand the operationalization and measurement of race, ethnicity, sexuality, sex, and gender, as well as SDOH, by using tools that have been standardized with health equity in mind. The PhenX Toolkit is one such tool that offers a variety of measures that experts have created across demographics and all levels of SDOH [77]. In addition, using standardized questions and responses, available data can be better used in future meta-analyses, further enhancing the potential understanding of LARC uptake and use with intersectional identities and multilayered SDOH. Systematic reviews and meta-analyses increase the accessibility of evidence [78]. Furthermore, by locating, assessing, and drawing out major themes and findings, they can provide essential information about current evidence and areas that need to be developed for decision-makers at all levels of practice [78]. Additionally, research that provides a more robust analysis of the barriers to adolescent LARC uptake can better inform future interventions to increase accessibility and self-determination.

## 5. Conclusions

LARCs can be an effective and accessible tool for adolescent and young adult birthing people who wish to prevent pregnancy, among other health and personal concerns. Interventions to promote LARC are needed to address SDOH barriers to access, including financial barriers, access to skilled and specialized providers, and education about efficacy. However, research evaluating LARC uptake and continuation must purposefully and accurately measure race, gender, sex, and sexuality. Minoritized populations face additional intersectional hurdles to reproductive autonomy that must be considered and measured in LARC research. Future research on LARC can leverage contemporary measurement tools for demographics and SDOH as well as qualitative work to allow participants to self-identify to ensure all racial, ethnic sexual, and gender and sex-expansive identities are included in the data used to design interventions, policy, and planning related to LARC use for young people and adolescents.

## Supplementary Material

Supplementary Material

## Figures and Tables

**Table 1.** Summary of study characteristics.

Study Name	First Author, Year	US State, BEA * Region	Study Design	Sample Size	Age Range	LARC Methods Examined	Source of Participants	Operationalization of Race and/or Ethnicity
Impact of the Rochester LARC Initiative on adolescents’ utilization of long-acting reversible contraception	Aligne, 2020	National in scope/Multiple regions	Quantitative	Not specifically listed though based on national sample sizes it would theoretically be combined 2013 and 2017 = 28,348	“High school students”	Defined as “LARC”	Secondary data from the national Youth Risk Behavior Surveillance System (YRBSS)	Race/Ethnicity: White; African-American; Hispanic or Latino
Retrospective Review of Intrauterine Device in Adolescent and Young Women	Alton, 2012	Kentucky; Southeast Region	Quantitative	233	menarche to 21 years	Hormonal IUD, Non-hormonal IUD	Pediatric and Adolescent gynecology private practice, a Title X clinic, and community based, grant funded clinic serving a high risk teen population.	Not reported
Will it Hurt? The Intrauterine Device Insertion Experience and Long-Term Acceptability Among Adolescents and Young Women	Callahan, 2019	Massachusetts; New England Region	Quantitative	95	13–21 years	Hormonal IUD, Non-hormonal IUD	Boston Children’s Hospital and Cambridge Health Alliance	Race: Black; White; Asian; Other; Not Reported; Ethnicity: Hispanic/Latino; Not Hispanic/Latino; Other
The Impact of an Adolescent Gynecology Provider on Intrauterine Device and Subdermal Contraceptive Implant Use Among Adolescent Patients	Crain, 2019	West Virginia; Southeast Region	Quantitative	2401	13–24 years	Hormonal IUD, Non-hormonal IUD, Subdermal contraceptive implant	Academic Practice and Title X funded patients	Not reported
Long-Acting Reversible Contraception Counseling and Use for Older Adolescents and Nulliparous Women	Gibbs, 2016	California, Colorado, Connecticut, Florida, Hawaii, Idaho, Michigan, Minnesota, New Jersey, New Mexico, North Carolina, Ohio, Oregon, Pennsylvania, and Washington; National in scope/multiple regions	Quantitative	1500	18–25 years	Hormonal IUD, Non-hormonal IUD, Subdermal contraceptive implant	Planned Parenthood health centers serving low-income, diverse patient populations	Race/Ethnicity: White; Hispanic; Black; Other
Follow-Up Care and 6-Month Continuation Rates for LongActing Reversible Contraceptives in Adolescents and Young Adults: A Retrospective Chart Review	Jones, 2020	Pennsylvania; Mideast Region	Quantitative	177	13–23 years	Hormonal IUD, Non-hormonal IUD, Subdermal contraceptive implant	Urban adolescent specialty care clinic	Race/Ethnicity: non-Hispanic White, non-Hispanic Black, Hispanic or Latino, Other
Intrauterine Contraception in Adolescents and Young Women: A Descriptive Study of Use, Side Effects, and Compliance	Lara-Torre, 2011	Virginia; Southeast Region	Quantitative	89	22 years or younger	Hormonal IUD, Non-hormonal IUD	An urban residency program OB/GYN clinic	Not labeled as Race or Ethnicity: Caucasian, African American, Hispanic
Acceptance of long-acting reversible contraceptive methods by adolescent participants in the Contraceptive CHOICE Project	Mestad, 2011	Missouri; Plains Region	Quantitative	5086	14–20 years	Hormonal IUD, Non-hormonal IUD, Subdermal contraceptive implant	University-affiliated clinics, two facilities providing abortion services, and community clinics that provide family planning, obstetric, gynecologic, and/or primary care	Race: Black, White, Other; Ethnicity: Hispanic (y/n)
Improving LARC Access for Urban Adolescents and Young Adults in the Pediatric Primary Care Setting	Onyewuchi, 2019	Maryland; Mideast Region	Quantitative	104	13–24 years	Hormonal IUD, Non-hormonal IUD, Subdermal contraceptive implant	University Pediatric Clinic	Race/ethnicity: Black, White, Hispanic, Other
Game change in Colorado: Widespread use of long-acting reversible contraceptives and rapid decline in births among young, low-income women. Perspectives on sexual and reproductive health	Ricketts, 2014	Colorado; Rocky Mountain Region	Quantitative	48,740	15–24 years	Hormonal IUD, Non-hormonal IUD, Subdermal contraceptive implant	Title X-funded Clinics	Race: White, Black, Asian/Pacific Islander, American Indian/Native Alaskan, Other, Unknown;
Vital Signs: Trends in Use of Long-Acting Reversible Contraception Among Teens Aged 15–19 Years Seeking Contraceptive Services—United States, 2005–201	Romero, 2015	National in scope/multiple regions	Quantitative	616,148	15–19 years	Hormonal IUD, Non-hormonal IUD, Subdermal contraceptive implant	Family Planning Annual Report, United States	Not Reported
Urban adolescents and young adults’ decision-making process around selecting intrauterine contraception	Rubin, 2016	New York; Mideast Region	Qualitative	27	16–25 years	Hormonal IUD, Non-hormonal IUD	Outpatient adolescent medicine clinic located within an academic children’s hospital	Ethnicity only: Latina
Integrating Long-Acting Reversible Contraception Services into New York City School-Based Health Centers: Quality Improvement to Ensure Provision of Youth-Friendly Services	Sangraula, 2016	New York; Mideast Region	Qualitative	18	15–19 years	Hormonal IUD, Non-hormonal IUD, Subdermal contraceptive implant	School Based Health Centers	Not measured
Promotion of Long-Acting Reversible Contraception Among Adolescents and Young Adults	Santibenchakul, 2019	Hawai’i; Far West Region	Quantitative	450 visits	14–25 years	Hormonal IUD, Non-hormonal IUD, Subdermal contraceptive implant	Obstetrics and Gynecology clinic	Race: Asian, Pacific Islander, White, Native Hawaiian, Other; Ethnicity: Hispanic or Latino; not Hispanic or Latino; not documented
Adolescent Experiences With Intrauterine Devices: A Qualitative Study	Schmidt, 2015	Missouri; Plains Region	Qualitative	43	14–19 years	Hormonal IUD, Non-hormonal IUD	University based clinic in the Contraceptive CHOICE pilot project	Race/Ethnicity: Latina, African American, White, Other
Pediatric Provider Education and Use of Long-Acting Reversible Contraception in Adolescents	Smith, 2019	Massachusetts; Mideast Region	Quantitative	7331	15–21 years	Hormonal IUD, Non-hormonal IUD, Subdermal contraceptive implant	Large health system	Not Reported
Provider and health system factors associated with usage of long-acting reversible contraception in adolescents.	Smith, 2017	Massachusetts; Mideast Region	Quantitative	5363	15–21 years	Hormonal IUD, Non-hormonal IUD, Subdermal contraceptive implant	Multiple sites (clinics, hospitals)	Not Reported
Adolescents’ Acceptance of Long-Acting Reversible Contraception After an Educational Intervention in the Emergency Department: A Randomized Controlled Trial	Vayngortin, 2020	California; Far West Region	Quantitative	79	14–21 years	Hormonal IUD, Non-hormonal IUD, Subdermal contraceptive implant	Urban pediatric emergency department	Ethnicity only: African-American, Hispanic, Multi-Ethnic, Caucasian, Asian/Pacific Islander
Use of the Intrauterine Device Among Adolescent and Young Adult Women in the United States From 2002 to 2010	Whitaker, 2013	National in scope/multiple regions	Quantitative	4684	15–24 years	Hormonal IUD, Non-hormonal IUD, Subdermal contraceptive implant	Secondary data from the National Survey of Family Growth (NSFG)	Race/Ethnicity: White/non-Hispanic, Black/non-Hispanic, Hispanic, Other/Multiracial

* BEA is the US Bureau of Economic Analysis.

**Table 2.** Social Determinants and LARC Use Identified in Included Studies versus Levels of Dahlgren and Whitehead Model.

Dahlgren and Whitehead Model Levels	Social Determinants
General socio-economic, cultural, and environmental conditions	Geographical area [52] Public LARC education [17] State with Medicaid expansion [51] Private funding for LARC [39,61,62] WIC usage in the area * [39] Internet and media [56]

Living and working conditions	Health services [39,51,52,54–62,64–67] Health insurance/payor status ** [39,51,53–56,61–64] Education [17,53,56,61,62] Income/Federal Poverty Level [39,53,61,62]
	
Social and community networks	Marital/partner status [51,53,61,64] Sex partner opinion/experience [56,60] Social support and influence [56,57] Religion [53]

Individual lifestyle factors	Substance use (e.g., tobacco, alcohol, drugs) [60] Sexual and reproductive factors [51,53,64,67]

Age, sex, and constitutional factors (nonbiological, physiologic, or genetic)	Age 17, [39,51–67] Sex 17, [39,51–67] Race and/or ethnicity [17,39,51,53–56,60–65] Biological [17,39,51,53–57,59–65,67] Born outside of the US [53]

* WIC is the Special Supplemental Nutrition Program for Women, Infants, and Children is an American federal assistance program of the Food and Nutrition Service of the United States Department of Agriculture for healthcare and nutrition of low-income pregnant women, breastfeeding women, and children under the age of five and was used a proxy for birth rates in low socio-economic status populations.

** Payor is an entity that pays for services by a healthcare provider, including employer-based health insurance, services paid or reimbursed by the military for service members or veterans, grant funding, or others.

## Data Availability

The data presented in this study are available in Figure 1, Table 1, and Supplementary Materials.

## Appendix A

Appendix A contains the Adapted JBI Critical Appraisal Checklist for Analytical Cross-Sectional Quality Assessment with specific questions used. Updated Checklist

1. Were the criteria for inclusion in the sample clearly defined?
2. Were the study subjects and the setting described in detail?
3. Was the social determinant of health measured in a valid and reliable way?
4. Were objective, standard criteria used for measurement of LARC uptake/insertion?
5. Were confounding factors identified?
6. Were strategies to deal with confounding factors stated?
7. Was LARC uptake/continuation clearly defined?
8. Were participants lost to follow up clearly described, (i.e. number withdrawn, reason for withdrawal)?
