# Supplementary material for: How Can We Address What We Do Not Measure? A Systematic Scoping Review of the Measurement and Operationalization of Social Determinants of Health Research on Long-Acting Reversible Contraceptive among Adolescents in the US
Source: Adolescents. Author manuscript; Available in PMC 2024 Jun 21. (PMC11192542; doi:10.3390/adolescents3020018)
Supplement: Supplementary Material [file NIHMS1971730-supplement-Supplementary_Material.pdf]

Supplementary 1 Search Strategy

PubMed- 12/16/2020- 991
((((((((((long acting reversible contraceptives[Title/Abstract]) OR (long-acting reversible contraceptives[Title/Abstract])) OR (long acting reversible contraception[Text Word])) OR (LARC[Title/Abstract])) OR (intrauterine device[Text Word])) OR (intrauterine devices[Text Word])) OR (IUD[Text Word])) OR (IUDs[Title/Abstract])) OR ((((((((paragard[Title/Abstract]) OR (mirena[Title/Abstract])) OR (kyleena[Title/Abstract])) OR (liletta[Title/Abstract])) OR (skyla[Title/Abstract])) OR (copper IUDs[Title/Abstract])) OR (hormonal IUDs[Title/Abstract])) OR (hormonal IUD[Title/Abstract])) OR (copper IUD[Title/Abstract]))) OR ((((((((((((((((subdermal contraceptive implants[Title/Abstract]) OR (subdermal contraceptive implant[Title/Abstract])) OR (subdermal implants[Title/Abstract])) OR (subdermal implant[Title/Abstract])) OR (contraceptive implant[Title/Abstract])) OR (contraceptive implants[Title/Abstract])) OR (birth control implant[Title/Abstract])) OR (birth control implants[Title/Abstract])) OR (progestin implant[Title/Abstract])) OR (progestin implants[Title/Abstract])) OR (etonogestrel implant[Title/Abstract])) OR (etonogestrel implants[Title/Abstract])) OR (progestin etonogestrel implant[Title/Abstract])) OR (progestin etonogestrel implants[Title/Abstract])) OR (ENG implant[Title/Abstract])) OR (ENG implants[Title/Abstract])) OR (nexplanon[Title/Abstract])) OR ((((((((jardelle) OR (implanon[Title/Abstract])) OR (norplant[Title/Abstract])) OR (sino-implant (ii)[Title/Abstract])) OR (levoplant TM[Title/Abstract])) OR (zarin[Title/Abstract])) OR (femplant[Title/Abstract])) OR (simplant[Title/Abstract])) OR (trust implant[Title/Abstract])))) OR ((((((((((Depo-Provera[Title/Abstract]) OR (contraceptive injection[Title/Abstract])) OR (contraceptive injections[Title/Abstract])) OR (birth control shot[Title/Abstract])) OR (birth control injection[Title/Abstract])) OR (medroxyprogesterone acetate[Title/Abstract])) OR (DMPA contraceptive injection[Title/Abstract])) OR (DMPA contraceptive injections[Title/Abstract])) OR (Depo-SubQ Provera 104[Title/Abstract])) OR (birth control shots[Title/Abstract])) OR (birth control injections[Title/Abstract]))) AND ((health disparities[sb]) OR (((((((("Insurance Coverage"[Mesh]) OR (insurance coverage[Title/Abstract])) OR (insurance status[Title/Abstract])) OR (insured[Title/Abstract])) OR (uninsured[Title/Abstract])) OR (underinsured[Title/Abstract])) OR (self pay[Title/Abstract])) OR (insurance type)))

Embase- 12/16/2020 1210 results
('long-acting reversible contraception'/exp OR 'long-acting reversible contraception' OR 'long acting reversible contraception':ti,ab OR 'long acting reversible contraceptives':ti,ab OR 'long acting reversible contraceptive':ti,ab OR 'intrauterine contraceptive device':ti,ab OR 'intrauterine contraceptive':ti,ab OR 'intrauterine device':ti,ab OR 'intrauterine devices':ti,ab OR 'iud':ti,ab OR 'iuds':ti,ab OR 'copper intrauterine device'/exp OR 'copper intrauterine device' OR 'levonorgestrel'/exp OR 'levonorgestrel' OR 'copper intrauterine device':ti,ab OR 'hormonal intrauterine device':ti,ab OR 'hormonal iud':ti,ab OR 'paragard':ti,ab OR 'mirena':ti,ab OR 'kyleena':ti,ab OR 'liletta':ti,ab OR 'skyla':ti,ab OR 'subdermal contraceptive implant'/exp OR 'subdermal contraceptive implant' OR 'birth control implant'/exp OR 'birth control implant' OR 'progestin implant'/exp OR 'progestin implant' OR 'etonogestrel implant'/exp OR 'etonogestrel implant' OR 'contraceptive implants'/exp OR 'contraceptive implants' OR 'contraceptive implant'/exp OR 'contraceptive implant' OR 'subdermal contraceptive implant':ti,ab OR 'birth control implant':ti,ab OR 'birth control implants':ti,ab OR 'progestin implant':ti,ab OR 'etonogestrel implant':ti,ab OR 'contraceptive implants':ti,ab OR 'contraceptive implant':ti,ab OR 'subdermal implant':ti,ab OR 'progestin etonogestrel implant':ti,ab OR 'progestin etonogestrel implants':ti,ab OR 'eng implant':ti,ab OR 'eng implants':ti,ab OR 'nexplanon':ti,ab OR 'jadelle':ti,ab OR 'implanon':ti,ab OR 'norplant':ti,ab OR 'sino-implant':ti,ab OR 'levoplant':ti,ab OR 'zarin':ti,ab OR 'femplant':ti,ab OR 'trust implant':ti,ab OR

'simplant':ti,ab OR 'medroxyprogesterone acetate'/exp OR 'medroxyprogesterone acetate' OR 'injectable contraceptive agent'/exp OR 'injectable contraceptive agent' OR 'injectable contraceptive agent':ti,ab OR 'contraceptive injection':ti,ab OR 'contraceptive injections':ti,ab OR 'birth control injection':ti,ab OR 'birth control shot':ti,ab OR 'birth control shots':ti,ab OR 'medroxyprogesterone acetate':ti,ab OR 'depo provera':ti,ab OR 'depoprovera':ti,ab OR 'dmpa contraceptive injection':ti,ab OR 'dmpa contraceptive injections':ti,ab OR 'depo-subq provera 104':ti,ab) AND ((('demography' OR 'environmental planning' OR 'marriage' OR 'divorce' OR 'cohabitation' OR 'widow' OR 'single marital status' OR 'neighbo*rhood*' OR 'residential environment*' OR 'rural*' OR 'innercity' OR 'inner city' OR 'housing instability' OR 'housing insecurity' OR 'housing strain' OR 'housing security' OR 'mortgage problems' OR 'foreclosure' OR 'eviction*' OR 'housing loss' OR 'home repossession*' OR 'home ownership' OR 'mortgage delinquency' OR 'mortgage arrears' OR 'mortgage debt*' OR 'overcrowding' OR 'marital status' OR 'marriage status' OR widow* OR cohabit* OR divorce* OR 'single parent*' OR 'live* alone' OR 'cultural deprivation' OR 'cultural factor' OR 'cultural anthropology' OR 'cultural diversity' OR 'migrant' OR 'minority group' OR 'minority health' OR 'prejudice' OR 'social discrimination' OR 'race relation' OR 'ethnic group' OR 'ancestry group' OR 'refugee' OR 'minorit*' OR 'migration background' OR 'racial' OR 'racism' OR 'ethnology' OR 'race' OR 'non*english' OR 'language other than' OR 'latino*' OR 'latina*' OR 'latin*' OR 'hispanic*' OR 'whites' OR 'caucasian*' OR 'non*white' OR 'aboriginal' OR 'native american' OR 'inuit' OR 'eskimo' OR 'first nation*' OR 'indigenous' OR 'english as a second language' OR 'foreign language' OR 'employment status' OR 'job characteristics' OR 'occupations' OR 'unemployment' OR 'gender identity' OR womens) AND health OR 'sex difference' OR 'sex disparit*' OR 'sex difference*' OR 'gender identity' OR 'sex role' OR 'wom?n* role*' OR 'm?n* role*' OR 'gender* role*' OR 'servicewomen' OR 'schooling' OR 'educational status' OR 'religion' OR 'religi*' OR 'social determinants of health' OR 'social aspect' OR 'working poor' OR 'social hierarchy' OR 'socioeconomics' OR 'disparit*' OR 'inequit* or (equity) or (deprivation) or (gini)' OR 'concentration index' OR 'social welfare' OR 'social class' OR 'social class*' OR 'social determinants' OR 'social status' OR 'social position' OR 'social background' OR 'social circumstance*' OR 'socio-economic' OR 'socioeconomic' OR 'sociodemographic' OR 'socio-demographic' OR 'ses' OR 'disadvantaged' OR 'impoverished' OR 'poverty' OR 'economic level' OR 'assets index' OR 'income*' OR 'social isolation' OR 'social capital' OR 'social stigma' OR 'social environment' OR 'social exclusion' OR 'social relationships' OR 'social network*' OR 'collective efficacy' OR 'civil society' OR 'informal social control' OR 'neighbo*rhood disorder' OR 'social disorgani*ation' OR 'anomie' OR 'social support' OR 'social participation' OR 'trust' OR 'emotional support' OR 'psychosocial support' OR 'community capital' OR 'neighbo*rhood cohesion' OR 'social influence' OR soci*context* OR 'soci* context*' OR 'health disparity' OR 'health equity' OR 'health care access' OR 'health*care disparit*' OR 'health care disparit*' OR 'health status disparit*' OR 'health disparit*' OR 'health inequality*' OR 'health inequit*' OR 'medically underserved' OR 'potential determinants' OR 'significant correlates of' OR ((independent AND correlates OR independent) AND association*) OR 'variables associated with' OR 'determinants of' OR 'factors associated with' OR 'identif* determinants' OR 'risk factors for' OR ((significantly AND related AND to OR significant) AND predictor) OR ((positively AND associated OR negatively) AND associated) OR 'differed by' OR ((were AND high* AND amongst OR were) AND low* AND amongst) OR (((inverse AND relationship AND with OR inversely) AND associated AND with OR inversely) AND related AND to) OR 'reverse association' OR 'differentially affects' OR 'evidence of a link between') AND (significant AND among OR no*) AND significant AND among OR 'health insurance'/exp OR 'insurance coverage':ti,ab OR 'health insurance':ti,ab OR 'insurance status':ti,ab OR 'insured':ti,ab OR 'uninsured':ti,ab OR 'underinsured':ti,ab OR 'self pay':ti,ab OR 'insurance type':ti,ab)
Web of science 12/16/2020 4152
(TS=(((ethnic dispari* or health disparit* or health care disparit* or culturally competent care or healthcare delivery or health behavio*r* or health inequ* or health services or health status disparit* or mass screening* or health care quality or social determinant* or social disparit* or social factor* or social inequ*

or socioeconomic factor* or socioeconomic disadvantage* or medically uninsured or underinsured or social discrimination or social marginalization or social segregation or underserved or urban health or urban population* or rural health or rural population* or teenager* or teen* or adolescent* or disab* or diverse population* or emigrant* or immigrant* or ethnic inequ* or ethnic population* or homeless or inmate* or jail or minorit* or migrant* or poverty or impoverished or prisoner* or race or racism or transient* or vulnerable or vulnerable
population*))) OR TS=(((black OR african american OR white OR caucasian OR asian american O R latin* OR hispanic OR mexican OR indigneous OR native american OR alaskan OR hawaiian))))
AND (TS=(long acting reversible contracept* or larc or intrauterine contracept* device* or IUD* or intrauterine device* or levonorgestrel or copper intrauterine device* or hormonal intrauterine device* or copper IUD* or hormonal IUD* or Paragard or Mirena or Kyleena or Liletta or Sklya or subdermal contracept* implant* or birth control implant* contracept* implant* or progestin implant* or etonogestrel implant* or progestin etonogestrel implant* or eng implant* or Nexplanon or Jadelle or Implanon or Norplant or sino-implant or levoplant or zarin or femplant or trust implant or simplant or medroxyprogesterone acetate or inject* contracept* or contraceptive inject* or birth control injection* or birth control shot or birth control shots or depo provera or depoprovera or dmpa contraceptive injection* or depo subq provera 104))

CINAHL- 12/16/2020 755

(TI (ethnic disparit* or health disparit*) OR AB (ethnic disparit* or health disparit*) OR TI (socioeconomic status or socioeconomic factor* or socioeconomic disadvantage*) OR AB (socioeconomic status or socioeconomic factor* or socioeconomic disadvantage*) OR TI cultural competenc* OR AB cultural competenc* OR TI health behavio#r* OR health behavio#r* OR TI (health inequalit* or health inequit* or social determinants of health) OR AB (health inequalit* or health inequit* or social determinants of health) OR TI (social disparit* or social class* or social factor* or social inequit*) OR AB (social disparit* or social class* or social factor* or social inequit*) OR TI (uninsured or medically uninsured or medically underinsured or medically underserved) OR AB (uninsured or medically uninsured or medically underinsured or medically underserved) OR TI (poverty or impoverished or low income) OR AB (poverty or impoverished or low income) OR TI (urban health or urban population*) OR AB (urban health or urban population*) OR TI (rural health or rural population*) OR AB (rural health or rural population*) OR TI (social discrimination or social marginalization or social segregation) OR AB (social discrimination or social marginalization or social segregation)) OR (TI (black or african american) OR AB (black or african american) OR TI (white or caucasian) OR AB (white or caucasian) OR TI asian american OR AB asian american OR TI (latin* or latin# or hispanic or mexican) OR AB (latin* or latin# or hispanic or mexican) OR TI (indigenous or native american or american indian or alaskan or hawaiian) OR AB (indigenous or native american or american indian or alaskan or hawaiian))

AND

TI (long acting reversible contraception or long acting reversible contraceptives or larc or intrauterine contraceptive devices or intrauterine devices or IUDs or levonorgestrel or copper intrauterine device or hormonal intrauterine device or copper IUD or hormonal IUD or paragard or mirena or kyleena or liletta or skyla) OR AB (long acting reversible contraception or long acting reversible contraceptives or larc or intrauterine contraceptive devices or intrauterine devices or IUDs or levonorgestrel or copper intrauterine device or hormonal intrauterine device or copper IUD or hormonal IUD or paragard or mirena or kyleena or liletta or skyla) OR TI (subdermal contraceptive implants or subdermal contraception implants or subdermal implants or birth contro limplants or contraceptive implants or etonogestrel implants or progestin implants or progestin etonogestrel implants or eng implants or nexplanon or jadelle or implanon or norplant or sino-implant or levoplant or zarin or femplant or trust implant or simplant) OR AB (subdermal contraceptive implants or subdermal contraception implants or

subdermal implants or birth contro limplants or contraceptive implants or etonogestrel implants or progestin implants or progestin etonogestrel implants or eng implants or nexplanon or jadelle or implanon or norplant or sino-implant or levoplant or zarin or femplant or trust implant or simplant) OR TI (medroxyprogesterone acetate or injectable contraceptives or injectable contraception or contraceptive injection or birth control injection or birth control shot or depo provera or depoprovera or dmpa contraceptive injection or depo subq provera 104) OR AB (medroxyprogesterone acetate or injectable contraceptives or injectable contraception or contraceptive injection or birth control injection or birth control shot or depo provera or depoprovera or dmpa contraceptive injection or depo subq provera 104)

PsycINFO- 12/16/2020- 518 results

(TI (ethnic disparit* or health disparit*) OR AB (ethnic disparit* or health disparit*) OR TI (socioeconomic status or socioeconomic factor* or socioeconomic disadvantage*) OR AB (socioeconomic status or socioeconomic factor* or socioeconomic disadvantage*) OR TI cultural competenc* OR AB cultural competenc* OR TI health behavio#r* OR health behavio#r* OR TI (health inequalit* or health inequit* or social determinants of health) OR AB (health inequalit* or health inequit* or social determinants of health) OR TI (social disparit* or social class* or social factor* or social inequit*) OR AB (social disparit* or social class* or social factor* or social inequit*) OR TI (uninsured or medically uninsured or medically underinsured or medically underserved) OR AB (uninsured or medically uninsured or medically underinsured or medically underserved) OR TI (poverty or impoverished or low income) OR AB (poverty or impoverished or low income) OR TI (urban health or urban population*) OR AB (urban health or urban population*) OR TI (rural health or rural population*) OR AB (rural health or rural population*) OR TI (social discrimination or social marginalization or social segregation) OR AB (social discrimination or social marginalization or social segregation)) OR (TI (black or african american) OR AB (black or african american) OR TI (white or caucasian) OR AB (white or caucasian) OR TI asian american OR AB asian american OR TI (latin* or latin# or hispanic or mexican) OR AB (latin* or latin# or hispanic or mexican) OR TI (indigenous or native american or american indian or alaskan or hawaiian) OR AB (indigenous or native american or american indian or alaskan or hawaiian))

AND

TI (long acting reversible contraception or long acting reversible contraceptives or larc or intrauterine contraceptive devices or intrauterine devices or IUDs or levonorgestrel or copper intrauterine device or hormonal intrauterine device or copper IUD or hormonal IUD or paragard or mirena or kyleena or liletta or skyla) OR AB (long acting reversible contraception or long acting reversible contraceptives or larc or intrauterine contraceptive devices or intrauterine devices or IUDs or levonorgestrel or copper intrauterine device or hormonal intrauterine device or copper IUD or hormonal IUD or paragard or mirena or kyleena or liletta or skyla) OR TI (subdermal contraceptive implants or subdermal contraception implants or subdermal implants or birth contro limplants or contraceptive implants or etonogestrel implants or progestin implants or progestin etonogestrel implants or eng implants or nexplanon or jadelle or implanon or norplant or sino-implant or levoplant or zarin or femplant or trust implant or simplant) OR AB (subdermal contraceptive implants or subdermal contraception implants or subdermal implants or birth contro limplants or contraceptive implants or etonogestrel implants or progestin implants or progestin etonogestrel implants or eng implants or nexplanon or jadelle or implanon or norplant or sino-implant or levoplant or zarin or femplant or trust implant or simplant) OR TI (medroxyprogesterone acetate or injectable contraceptives or injectable contraception or contraceptive injection or birth control injection or birth control shot or depo provera or depoprovera or dmpa contraceptive injection or depo subq provera 104) OR AB (medroxyprogesterone acetate or injectable contraceptives or injectable contraception or contraceptive injection or birth control injection or birth control shot or depo provera or depoprovera or dmpa contraceptive injection or depo subq provera 104)
